# Rising Levels of HIV Infection in Older Adults in Eastern Zimbabwe

**DOI:** 10.1371/journal.pone.0162967

**Published:** 2016-11-09

**Authors:** Joel Negin, Simon Gregson, Jeffrey W. Eaton, Nadine Schur, Albert Takaruza, Peter Mason, Constance Nyamukapa

**Affiliations:** 1 School of Public Health, University of Sydney, Sydney, Australia; 2 Biomedical Research and Training Institute, Harare, Zimbabwe; 3 Department of Infectious Disease Epidemiology, Imperial College School of Public Health, London, United Kingdom; 4 University of Zimbabwe College of Health Sciences, Harare, Zimbabwe; Azienda Ospedaliera Universitaria di Perugia, ITALY

## Abstract

**Background:**

With the scale-up of antiretroviral treatment across Africa, many people are living longer with HIV. Understanding the ageing of the HIV cohort and sexual behaviour among older adults are important for appropriately responding to the changing demographics of people living with HIV.

**Methods:**

We used data from a large population-based open cohort in eastern Zimbabwe to examine HIV prevalence trends and incidence among those aged 45 years and older. Five survey rounds have been completed between 1998 and 2011. Incidence was analysed using midpoint between last negative and first positive HIV test.

**Results:**

Across the survey rounds, 13,071 individuals were followed for 57,676 person years. While HIV prevalence among people aged 15–44 has fallen across the five rounds, HIV prevalence among those aged 45–54 has increased since the 2006–08 survey round. In the 2009–11 round, HIV prevalence among men aged 45–54 was 23.4% compared to 11.0% among those aged 15–44. HIV positive people aged 45–54 now represent more than 20% of all those living with HIV in Manicaland. Among those aged 45 years and older, there were 85 seroconversions in 11,999 person years for an HIV incidence of 0.708 per 100 person years. Analysis of cohort data and assessment of behavioural risk factors for HIV infection among older people shows significantly lower levels of condom use among older adults and a number of seroconversions past the age of 50.

**Conclusions:**

The cohort of people living with HIV is ageing in Zimbabwe and the behaviour of older adults puts them at risk of HIV infection. Older adults must be included in both HIV prevention and treatment programs.

## Background

With large increases in the number of people accessing life-prolonging antiretroviral treatment (ART) [1], many more people with HIV in sub-Saharan Africa (SSA) are expected to live to older ages. Mathematical model estimates suggest that the number of people aged 50 years and older living with HIV has increased with an estimated 2.5 million older adults living with HIV in SSA [2,3]. UNAIDS released a special report on HIV and ageing in 2013 [4] and included older adults as a group of people “left behind” in the 2014 “Gap Report” [5]. In recent years, there has been increased research in this area on topics ranging from treatment outcomes [6] and prevention messaging [7,8] to stigma [9] and co-morbidities [10,11].

Despite the increased attention, there is still only limited empirical evidence on the extent of HIV prevalence in older age-groups in SSA populations. In particular, few studies have been published on the ageing of cohorts of people living with HIV (PLHIV) and, especially, that compare trends before and after the large-scale roll-out of ART. The few studies that do exist are derived from South African data [12–14] leaving the rest of the continent under-studied.

Similarly, whilst mathematical models estimate that 74,000 older adults in SSA are infected with HIV each year [5], empirical data on incidence and sexual risk behaviour among older people are largely lacking. A UNAIDS report stated that “it is possible that the rate of new HIV infections among people 50 years and older is higher than previously thought, but there is very little quantitative research into the sexual behaviours and HIV incidence among this age-group in sub-Saharan Africa” [4].

Using 14 years of longitudinal data from eastern Zimbabwe, we aimed to examine the ageing of the population of PLHIV before and after the introduction of ART and to understand the behavioural risk factors for HIV infection in older people. Zimbabwe’s HIV prevalence in 2013 remained high at 15.0% (plausibility bounds: 14.2%-15.7%) [5] despite having reduced prevalence and new infections dramatically over the past 15 years [15,16]. Manicaland Province in Zimbabwe has a population of approximately 1.6 million people and an HIV prevalence of 14.5%.

## Methods

The Manicaland HIV/STD Prevention Project (Manicaland survey) is a population-based open cohort survey conducted in 12 geographically distinct sites in Manicaland. We report data from five phased rounds of the survey conducted approximately every two to three years from 1998 to 2011. In each survey round, all residents of the study areas are enumerated in a household-based census, and eligible adults are invited to join the study. In the first two rounds (1998–2000 and 2001–2003), males aged 17–54 years and females aged 15–44 years were eligible to participate. In these two rounds, where more than one member of a marital grouping was resident in a household, one member of this marital group was selected at random to participate in the cohort. In the third (2003–2005) and subsequent rounds, the eligible age-ranges were extended to 15–54 years for both sexes. Due to funding constraints, in the two most recent rounds (2006–2008 and 2009–2011), participation in the cohort was limited to individuals from a randomly-selected two-thirds of households in each site (data available in S1 Fig). Participation rates ranged between 79.1% in round 5 and 87.5% in round 2. Most of the non-participation was due to eligible respondents being absent from home during survey visits; refusal rates amongst those found at home were low (<5%) in all rounds. Written informed consent was obtained from all participants and from parents / guardians of the minors included in the study.

Survey participants completed a 1–2 hour-long interview covering a range of areas including background characteristics, sexual behaviour, and knowledge and attitudes about HIV. Dried blood spots were taken and tested for HIV in an offsite laboratory using the COMBAIDS-RS HIV 1+2 Immunodot Assay (Span Diagnostics, India), with new sero-conversions confirmed using Vironostika HIV Uni-form II Plus O (Biomérieux, France).

Ethical approval for the Manicaland survey was provided by the Medical Research Council of Zimbabwe and the Imperial College Research Ethics Committee, London. More information on the Manicaland survey including the questionnaire used in this study is available in other project publications [15,17,18] and from the project website (http://www.manicalandhivproject.org/).

For the purposes of this study, we define an older adult as a person aged 45 years and above. While we acknowledge that current convention is to use 50 as the cut-off for older adults [4,5], we use 45 in this analysis due to the low age cut-off employed in early rounds of the Manicaland survey.

The national ART program started in April 2004 and reached about 8000 people by the end of that year. The number of Zimbabweans on ART increased to 99,500 in 2007 and 218,600 by the end of December 2009 (56% of those needing treatment). We determined survey data collected 1998–2005 representing the pre-ART period and 2006–2011 representing the post-ART period.

For the incidence estimates, entry (into the incidence calculation) is considered to be the individual’s first negative HIV test in the study. Exits (from the incidence calculation) are determined to be either at the date of the last negative HIV test or upon observed sero-conversion. The precise date of sero-conversion is not observed, but it must lie between the dates of the last negative test and the first positive test. Two methods have been used to impute dates of sero-conversion in incidence studies. In some cohort studies where HIV testing is only conducted periodically, a randomly imputed date between the last negative and first positive test has been used as the date of sero-conversion [19–21]. In the Manicaland survey, where data collection for the 12 sites is phased and, therefore, each round is spread over a period of 18–24 months, HIV testing is conducted almost continuously. Therefore, we use the midpoint between last negative and first positive test as the date of sero-conversion. This method has been used in a number of other incidence studies [22–25]. Poisson regression was used to calculate incidence.

Sexual behaviour variables including number of sexual partners, condom use and circumcision were analysed comparing males aged 15–44 to those aged 45 and older and females aged 15–44 to older females. Statistical analyses were conducted using SAS 9.4 (Cary, NC, USA).

## Results

**Fig 1:**
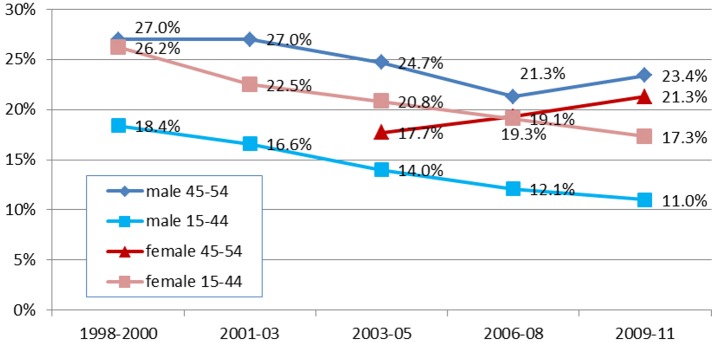
Comparison of trends in HIV prevalence among men and women aged 45–54 years and 15–44 years, by survey round.

Across the five survey rounds, HIV prevalence among men aged 15–44 declined steadily from 18.4% in 1998–2000 to 11.0%in 2009–2011 (P<0.0001) (Fig 1). HIV prevalence among men aged 45–54 also declined from 27.0% in 1998–2000 to 21.3% in 2006–2008 but then increased significantly (P = 0.001) to 23.4% in 2009–2011 (more than double the level amongst younger adults at this time). HIV prevalence in women aged 15–44 declined significantly (P<0.0001) from 20.8% in 2003–2005 to 17.3% in 2009–2011 but increased from 17.7% to 21.3% in older women over the same period.

**Fig 2:**
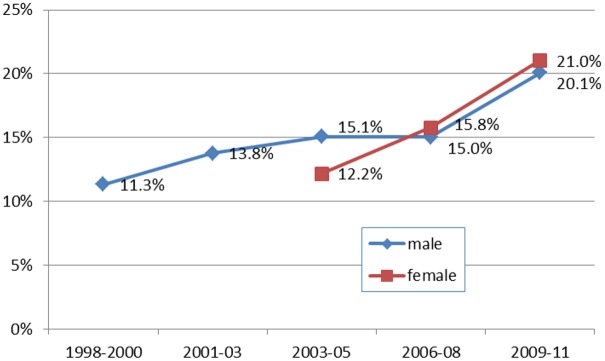
Percentage of People Living with HIV 15–54 years who are aged 45–54, by sex and survey round.

Along with increases in HIV prevalence among older adults, the proportion of all people aged 15–54 living with HIV who are aged 45–54 years has increased considerably. Whilst, in 1998–2000, only 11.3% of men living with HIV in the cohort were aged 45–54, just over 20% were of older age in the latest survey round (Fig 2) (P<0.0001). Among women living with HIV aged 15–54 years, the proportion aged 45–54 years rose steeply from 12.2% in 2003–2005 to 21.0% in 2009–2011 (P<0.0001).

With regard to the contribution of new infections to the ageing of the PLHIV cohort, over the fourteen year time period 1998 to 2011, there was a total of 57676 person years among 13,071 individuals for an average of 4.41 years per person. Across the rounds, 710 individuals have been followed up for more than 11 years.

Among older adults aged 45 and older, there were 89 sero-conversions in 11999 person years. HIV incidence among men aged 45 years and older was 1.03 per 100 person years which was non-significantly (P>0.05) lower than that of younger men 15–44 (1.27 per 100 person years) (Table 1). In the pre-ART time period of 1998–2005, HIV incidence among older men was the same as among younger men. HIV incidence among older women was less than half that of younger women.

**Table 1:** HIV incidence by age and pre- and post-treatment time period.

Sex	Year	Age	Sero-conversions	Person Years	Incidence Rate per 100 person years
Male	1998–2005	15–44	164	10632	1.54
		45+	29	1826	1.59
	2006–2011	15–44	72	8080	0.89
		45+	9	1777	0.51
	Total	15–44	236	18712	1.27
		45+	38	3603	1.03
Female	1998–2005	15–44	229	15294	1.5
		45+	26	3351	0.78
	2006–2011	15–44	156	11671	1.34
		45+	25	5045	0.5
	Total	15–44	385	26965	1.44
		45+	51	8396	0.57

Note: number of respondents per cell is available in S1 Table.

HIV incidence among all groups declined in the post-ART period (2006–2011) with both younger and older adults seeing similar declines in incidence.

The proportion of all new infections occurring in women aged 45 years and older in the 2006–2011 period was 13.8% which represents an increase from 10.2% in the 1998–2005 period. Conversely, among men, while 15.0% of new infections from 1998–2005 were among those aged 45 years and older, in the 2006–2011, 11.1% of new infections were among older men.

Limited data were available on the date of sero-conversion. Of the 528 people aged 45 and older living with HIV at survey round 5 (2009–2011), age at sero-conversion (and therefore an earlier negative HIV test) could be ascertained for 206 (39%). Of these 206, 55 (26.7%) sero-converted at age 45 or older. This limited data (focused on those included in survey round 5) suggest that there is a considerable amount of sero-conversion occurring among older adults.

Lastly, assessment of behavioural risk factors for HIV infection among older people shows that, for some behaviours, older adults are at lower risk than younger adults, but for other behaviours, they exhibit greater risk. Women aged 45 years and older were significantly more likely to have more than one regular partner than women aged 15–44 (4.2% versus 3.3%, P = 0.002) while both older men and women were significantly less likely to have used condoms throughout last sexual encounter (Table 2). At the same time, older men were more likely to be circumcised and older men and women had fewer partners in the last year than younger adults.

**Table 2:** Sexual behaviour of those aged 15–44 and those aged 45 and older by sex, round 5 only.

	MALE			FEMALE		
	15–44	45+	P	15–44	45+	P
Male circumcision	2.7%	6.4%	<0.001			
Ever married	49.4%	99.1%	<0.001	73.5%	98.9%	<0.001
Ever had a marital partner pass away	4.7%	14.3%	<0.001	12.8%	38.1%	<0.001
Percentage with more than one regular partner at time of survey	3.5%	4.8%	0.145	3.3%	4.2%	0.002
Condom use throughout last sex	25.1%	16.9%	<0.001	15.9%	13.5%	0.012
Ever accessed treatment for HIV	18.8%	6.7%	0.27	13.0%	22.0%	0.17
Mean days since last sex	25.1	19.1	0.001	33.5	54.3	<0.001
Mean number of sexual partners in last year	1.0	0.7	<0.001	0.8	0.5	<0.001
Mean times tested for HIV in last 3 years	1.3	1.2	0.078	1.6	1.4	<0.001

Note: number of respondents per cell is available in S2 Table.

## Discussion

In eastern Zimbabwe, HIV prevalence among people aged 45 and older is increasing at the same time that prevalence among younger age groups is decreasing. Older adults comprise an increasingly large proportion of those living with HIV in the region. At the same time, these older adults exhibit a range of behaviours that place them at risk for HIV infection including low levels of condom use and relatively higher rates of multiple partnerships. While HIV incidence among those aged 45 and older is lower than among those aged 15–44, there is evidence that as much as a quarter of older adults living with HIV may have seroconverted after the age of 45.

These findings are likely to reflect the reductions in HIV incidence—due to saturation of infection in high risk groups, behaviour change, and, most recently, a possible effect of widespread ART on transmission—and increased survival of PLHIV due to ART [15,16]. Given that ART has been shown to restore near-normal life expectancy [26], it is likely that such increases in older adults as a proportion of total PLHIV will be seen across SSA.

Our analysis however highlights that the increase in prevalence among older adults cannot be fully attributed to longer survival. Sero-conversion among older adults is occurring and the data presented here suggest that a number of older adults are undertaking behaviours that put them at risk of HIV transmission. A considerable proportion of older adults living with HIV sero-converted when already older—a fact that has not been sufficiently addressed in the literature.

There are a number of possible reasons for incidence among older adults. Older adults have been shown to have less knowledge about HIV and HIV transmission [7]. Assumptions about older adults not engaging in sexual behaviour [15] have limited the reach and relevance of targeted prevention messaging. Additionally, the thinning of the vaginal wall after menopause increases the chances of lesions thus increasing risk of HIV transmission during sex [27]. Lastly, it is possible that as the partners of those aged 50 and older die, older adults seek new relationships that put them at increased risk of HIV transmission.

Other studies have used models to estimate HIV incidence among older adults. The Kenyan government’s HIV update report from 2012 estimates that 5,000 to 15,000 older people were newly infected in 2011 out of a total of 91,000 new infections [28]. The Kenyan model thus estimates that 5.5% to 16.5% of new infections are among those aged 50 and older. Our study using longitudinal survey data, 13.0% of new infections are among those aged 45 and older. A similar longitudinal study in Uganda found higher HIV infection rates among those aged 40–59 than in those aged 30–39 [29]. The study also revealed that median age at HIV sero-conversion was substantially higher among men (40 years) than among women (28 years).

### Limitations

With 14 years of follow up data through five multi-year rounds of research, this is one of the longer periods of incidence study in an African general population sample. Many other incidence studies have used data from shorter periods [19,25,30,31]. However, it should be noted that those involved in long-term cohort studies might have had more exposure to HIV programmes including prevention and treatment efforts [32], thus potentially biasing the data though there is no direct evidence of that from previous Manicaland studies. In addition, the cohort is more likely to have data on long-term residents of the region rather than those who have moved in and out which creates a potential bias when incidence analyses rely on repeat inclusion in surveys.

Furthermore, the conclusions on age at sero-conversion are based on a relatively limited number of people for whom sufficient data was available; we could not impute age at sero-conversion for those who entered survey rounds already HIV-positive. Lastly, the fact that women up to age 44 only were included in the first two rounds and that age 55 was used as the cut-off subsequently reduces the pool of older adults able to be included in the survey. We acknowledge that people older than 54 are still sexually active and therefore at risk of HIV acquisition [8].

### Conclusions

These data confirm that older adults need to be included in prevention and treatment programmes in Manicaland and likely elsewhere in SSA with the possibility of specifically targeted programmes where service gaps are found. Other work from eastern Zimbabwe has highlighted the critical role of community groups in instilling change and promoting positive behaviour [18,33]. Providing sensitive support for some of these groups might be one approach to promoting HIV control amongst older adults. In addition, efforts are needed to ensure that testing and treatment services are responsive to the needs of older adults to encourage higher rates of service coverage [7,34].

## Supporting Information

S1 Fig Number of households reached in rounds 4 and 5. (DOCX)

S1 Table Numbers responding for HIV prevalence among men and women aged 45–54 years and 15–44 years, by survey round. (DOCX)

S2 Table Sexual behaviour of those aged 15–44 and those aged 45 and older by sex, round 5 only. (DOCX)
