# Supplementary material for: Rising Levels of HIV Infection in Older Adults in Eastern Zimbabwe
Source: PLoS One. 2016 Nov 9;11(11):e0162967. doi: 10.1371/journal.pone.0162967 (PMC5102380; doi:10.1371/journal.pone.0162967)
Supplement: S1 Fig — (DOCX) [file pone.0162967.s001.docx]

***S1 Fig. Number of households reached in rounds 4 and 5***

In round 4, individuals were selected from 7764/12661 households. In round 5, individuals were selected from 8837/13453 households.
