# Supplementary material for: Rising Levels of HIV Infection in Older Adults in Eastern Zimbabwe
Source: PLoS One. 2016 Nov 9;11(11):e0162967. doi: 10.1371/journal.pone.0162967 (PMC5102380; doi:10.1371/journal.pone.0162967)
Supplement: S1 Table — (DOCX) [file pone.0162967.s002.docx]

***S1 Table. Numbers responding for HIV prevalence among men and women aged 45-54 years and 15-44 years, by survey round***

|  | 1998-2000 | 2001-03 | 2003-05 | 2006-08 | 2009-11 |
| --- | --- | --- | --- | --- | --- |
| male 45-54 | 341 | 289 | 587 | 456 | 559 |
| male 15-44 | 3953 | 2944 | 5841 | 4538 | 4793 |
| female 45-54 |  |  | 1346 | 1149 | 1479 |
| female 15-44 | 4927 | 4157 | 8366 | 6214 | 6884 |
