# Supplementary material for: Rising Levels of HIV Infection in Older Adults in Eastern Zimbabwe
Source: PLoS One. 2016 Nov 9;11(11):e0162967. doi: 10.1371/journal.pone.0162967 (PMC5102380; doi:10.1371/journal.pone.0162967)
Supplement: S2 Table — (DOCX) [file pone.0162967.s003.docx]

***S2 Table. Sexual behaviour of those aged 15-44 and those aged 45 and older by sex, round 5 only***

|  | MALE | | FEMALE | |
| --- | --- | --- | --- | --- |
|  | 15-44 | 45+ | 15-44 | 45+ |
| Male circumcision | 4797 | 748 |  |  |
| Ever married | 4829 | 754 | 6887 | 1943 |
| Ever had a marital partner pass away | 2385 | 746 | 5315 | 1918 |
| Percentage with more than one regular partner at time of survey | 2206 | 672 | 4262 | 1095 |
| Condom use throughout last sex | 2894 | 753 | 5487 | 1936 |
| Mean days since last sex | 2896 | 754 | 5495 | 1937 |
| Mean number of sexual partners in last year | 2891 | 752 | 5483 | 1927 |
| Mean times tested for HIV in last 3 years | 1378 | 295 | 4323 | 1138 |
